# A structured interview guide for global impressions: increasing reliability and scoring accuracy for CNS trials

**DOI:** 10.1186/1744-859X-12-2

**Published:** 2013-01-31

**Authors:** Steven D Targum, Celine Houser, Joanne Northcutt, Jessica A Little, Andrew J Cutler, David P Walling

**Affiliations:** 1 Clintara LLC, 505 Tremont Street, Boston, MA, 02116, USA; 2 Florida Clinical Research Center, LLC 2300 Maitland Center Parkway, Suite 230, Maitland, FL, 32751, USA; 3 Collaborative Neuroscience Network, 12772 Valley View Street, Suite 3, Garden Grove, CA, 92845, USA

**Keywords:** Inter-rater reliability, Global impressions, Ratings accuracy, Structured interviews

## Abstract

**Background:**

The clinical global impression of severity (CGI-S) scale is a frequently used rating instrument for the assessment of global severity of illness in Central Nervous System (CNS) trials. Although scoring guidelines have been proposed to anchor these scores, the collection of sufficient documentation to support the derived score is not part of any standardized interview procedure. It is self evident that the absence of a standardized, documentary format can affect inter-rater reliability and may adversely affect the accuracy of the resulting data.

**Method:**

We developed a structured interview guide for global impressions (SIGGI) and evaluated the instrument in a 2-visit study of ambulatory patients with Major Depressive Disorder (MDD) or schizophrenia. Blinded, site-independent raters listened to audio recorded SIGGI interviews administered by site-based CGI raters. We compared SIGGI-derived CGI-S scores between the two separate site-based raters and the site-independent raters.

**Results:**

We found significant intraclass correlations (p = 0.001) on all SIGGI-derived CGI-S scores between two separate site-based CGI raters with each other (r = 0.768) and with a blinded, site-independent rater (r = 0.748 and r = 0.706 respectively) and significant Pearson’s correlations between CGI-S scores with all MADRS validity comparisons for MDD and PANSS comparisons for schizophrenia (p- 0.001 in all cases). Compared to site-based raters, the site-independent raters gave identical “dual” CGI-S scores to 67.6% and 68.2% of subjects at visit 1 and 77.1% at visit 2.

**Conclusion:**

We suggest that the SIGGI may improve the inter-rater reliability and scoring precision of the CGI-S and have broad applicability in CNS clinical trials.

## Background

Global measures of illness severity go beyond the quantitative scoring of symptom severity to weigh the clinical impact of the identified symptoms on behavior and function. The Clinical Global Impressions of severity (CGI-S) scale is a well-known and relatively straightforward single-item instrument used to assess the overall (global) severity of illness as a graded measure of increasing psychopathology from 1 to 7 [1]. The original description of the CGI-S provided the progressive seven-point range of scores but did not offer scoring anchors to standardize scoring between raters. Recently, published scoring guidelines have improved both inter-rater reliability and the precision of CGI scoring [2-4]. Specific versions of the CGI-S have been developed to address the unique symptoms and functional impact on illness for Bipolar disorder, Schizophrenia, Alzheimer’s disease, Obsessive-compulsive disorder, as well as for complex symptoms like fatigue [2,5-10]. Although these operational guidelines can facilitate scoring precision by differentiating between the seven progressive global severity scores, there is no standardized, interview procedure used to document and support these derived scores. It is self evident that the absence of a standardized, documentary format limits inter-rater reliability, may affect the validity of the resulting data, and may adversely affect the trial outcome [11-13]. Consequently, we have developed a structured interview guide for global impressions (SIGGI) to assist in the documentation of the global impression assessment. Although specific queries are provided to identify the relevant, acute symptoms and assess the clinical relevance of these symptoms, the SIGGI stays true to the original intent of the CGI to provide a global impression of illness based primarily on the clinician’s sound judgment and experience with a particular patient population.

In this paper, we describe the development of the SIGGI and results from a reliability and validity study conducted in patients with Major Depressive Disorder (MDD) and schizophrenia. There were two study objectives: 1) to assess the reliability and validity of the SIGGI in two distinct psychiatric populations, and 2) to explore the utility of audio-digital pen recorders in the “dual” assessment of CNS patients. We found a significantly high inter-rater agreement between SIGGI-derived CGI-S scores generated by site-based interviews and blinded, site-independent ratings based upon audio-recordings of these SIGGI interviews. We believe that the SIGGI is a reliable and valid instrument that standardizes the documentation of the global severity of illness assessment. We believe that the SIGGI may have broad applicability in CNS clinical trials.

## Methods

We evaluated the utility of the Structured Interview Guide for Global Impressions (SIGGI) in a two-visit study of psychiatric patients visiting 5 different clinical trial sites within the United States (Florida Clinical Research Center, Bradenton, Florida; FutureSearch Trials, Austin and Dallas, Texas; Pacific Research Partners, Oakland, California; Collaborative Neuroscience Network, Garden Grove, California). All sites obtained IRB approval to conduct this study. The objective was to evaluate a broad group of ambulatory subjects who had a range of illness severity with diagnoses of either Major Depressive Disorder (MDD) or schizophrenia. These patients were not enrolled in a clinical trial at the time of their participation in this study and no new treatments were offered between visits. Patients gave written, informed consent to participate in a survey of the impact of their current symptoms on their behavior and function. 71 subjects consented to participate in the study.

There is a well-established precedent for the development of structured interview guides to improve inter-rater reliability and the precision of psychiatric assessments [14-16]. The SIGGI is a semi-structured instrument that can be administered in approximately 10 minutes that was designed to improve scoring precision by requiring specific documentation to support the derived CGI severity scores. The SIGGI addresses three principal areas:

1) Relevant symptom identification

2) Documentation of *current* clinical relevance (impact) of the identified symptoms on behavior and function;

3) Identification of possible confounding factors that might influence or obscure accurate CGI-S scoring.

In previous studies, we have reported that confounding factors (e.g*.* exposure to recent trauma, losses, relocations) may adversely influence CGI scoring [4]. Raters provide written responses for each query and use the resulting documentation to derive the CGI-S score using appropriate scoring guidelines for the designated illness. Scoring guidelines emphasize the importance of assessing the impact of current, relevant clinical symptoms on behavior and function. Although the queries that compose the SIGGI can be customized for a specific clinical study, a sample template of the SIGGI interview is attached in Additional file 1: Appendix 1.

Nine trained site-based raters from the 5 trial sites used an audio-digital pen recorder to administer the SIGGI interview and score the CGI-S. This recording method provided a relatively unobtrusive site-independent strategy to generate a second “dual” score of the same subject without any informational variance because it was based entirely upon the site-based interview [17,18]. To assess reliability, the recorded interviews were electronically transmitted and scored by one of two site-independent raters (SDT, JAL) who were blinded to the patient’s diagnosis, site location, study visit, and all site-based scoring. As an additional assessment of inter-rater reliability, a second site-based rater administered and recorded the SIGGI interview with the same subjects at visit 1. A random sample of 50% of the subjects attending visit 1 were asked to return for a second, follow-up interview after 4–6 weeks (visit 2).

Both the site-based and site-independent raters scored the CGI-S using guidelines and scoring anchors that have previously been published [2-4].

Symptom severity was evaluated using either the Montgomery-Asberg Depression Rating Scale (MADRS) in the subjects with MDD, or the Positive and Negative Syndrome Scale (PANSS) in patients with schizophrenia [2,19]. These are reliable and validated instruments used to assess and quantify symptom severity in these patient populations and known to be correlated with the CGI-S [3,9,20].

Reliability of the SIGGI was established by intra-class correlation analysis ICC of the two site-based and the blinded, site-independent CGI-S scores. Validity was established by Pearson’s correlation of the CGI-S with either the MADRS or PANSS as appropriate in the designated patient population. We also examined the proportion of matched (identical) CGI-S scores generated by different raters on the same subject.

## Results

71 subjects (46 men and 25 women) attending 5 different clinical trial sites participated in visit 1 and 35 subjects returned for visit 2. At visit 1, 29 subjects met DSM-IV criteria for MDD and 42 subjects met criteria for schizophrenia. The subjects ranged in age from 22 to 64 years (mean age = 47.6 years ± 9.5 (SD). Based upon the elapsed time recorded by the audio-digital pen, the mean time of administration of the SIGGI interview after consent was 9.6 ± 8.65 (SD) minutes and ranged from 8 to 15 minutes among these patients.

**Figure 1:**
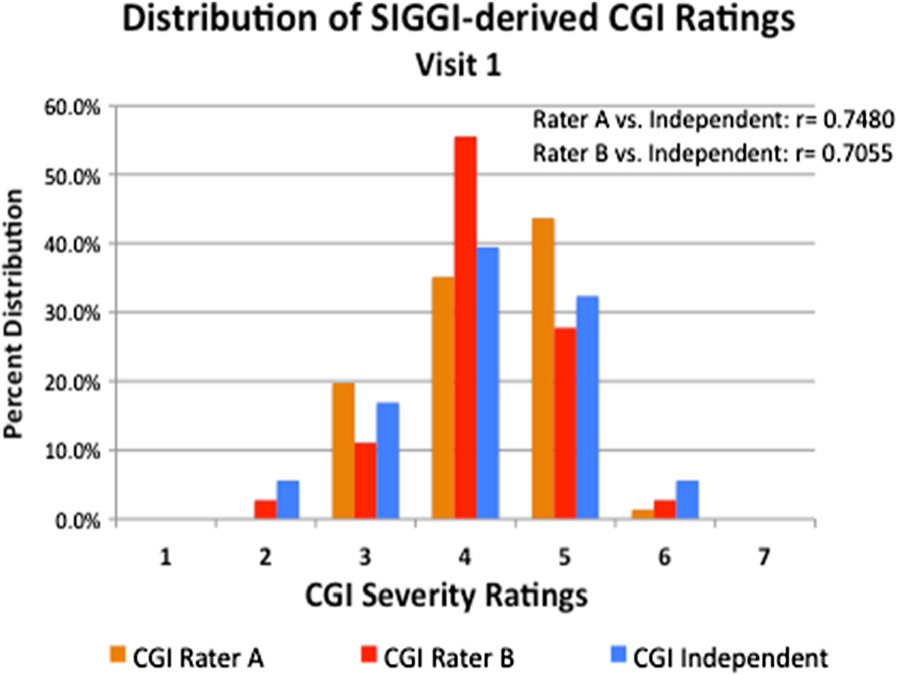
Distribution of SIGGI-Derived CGI-Severity Ratings: Visit 1.

**Figure 2:**
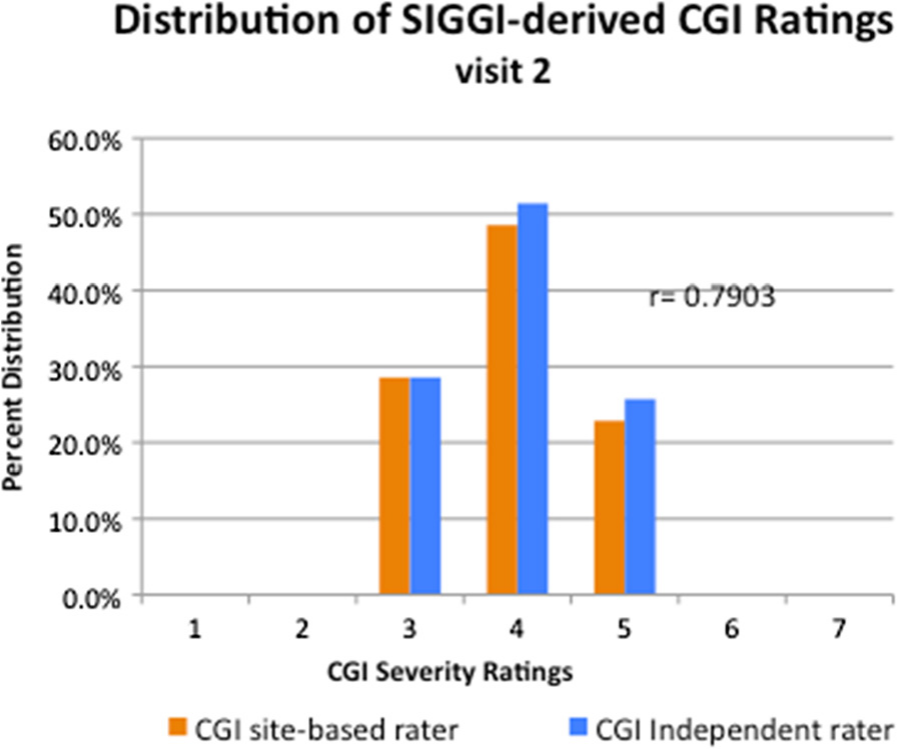
Distribution of SIGGI-Derived CGI-Severity Ratings: Visit 2.

At visit 1, the SIGGI-derived CGI-S scores ranged from 2 (borderline severity) to 6 (severe pathology), and narrowed to 3 to 5 at visit 2. The score distribution was similar between the site-based CGI raters and the site-independent rater at each visit (Figures 1 and 2). There were no statistically significant mean CGI-S scoring differences between the two site-based raters or between the site-based raters and the blinded, site-independent CGI rater on any measure (see Table 1).

**Table 1:** Summary of ratings: CGI-Severity, MADRS, PANSS

	**CGI-A rater**	**CGI-B rater**	**Independent**
**ALL SUBJECTS**			
visit 1 CGI-S (n = 71)	4.27 ± 0.79	4.17 ± 0.77	4.15 ± 0.97
visit 2 CGI-S (n = 35)	4.03 ± 0.75		3.91 ± 0.70
**MDD**			
visit 1 CGI-S (n = 29)	4.55 ± 0.63	4.46 ± 0.66	4.76 ± 0.69
visit 2 CGI-S (n = 6)	4.00 ± 0.63		3.83 ± 0.75
visit 1 MADRS	31.08 ± 7.20		
visit 2 MADRS	22.17 ± 9.28		
**schizophrenia**			
visit 1 CGI-S (n = 42)	4.07 ±0.84	4.00 ±0.80	3.74 ±0.91
visit 2 CGI-S (n = 29)	4.03 ± 0.78		3.97 ±0.70
visit 1 PANSS	67.52 ± 13.95		
visit 2 PANSS	70.62 ± 13.33		

At visit 1, there was a highly significant correlation between the blinded site-independent CGI-S ratings with site-based rater 1 (r = 0.748; p =0.001) and rater 2 (r = 0.706; p = 0.001) and between the two site-based CGI raters with each other (r = 0.768; p = 0.001).

Similarly, there was a high correlation at visit 2 between the site-based CGI rater and the site-independent rater (r = 0.790; p = 0.001).

We examined the proportion of matched (identical) agreement achieved on CGI-S scores for the same subject between the 2 site-independent raters and the 9 site-based raters.

Site-independent raters listened to the audiotaped SIGGI interviews to generate their blinded CGI-S scores. At visit 1, 48 of the 71 “dual” CGI-S scores (67.6%) between site-based rater’s A (first interview) and the site-independent ratings were identical, 31.0% were one point apart, and one paired CGI-S was two points apart (1.4%). Similarly, at visit 1 70.6% of “dual” CGI-S scores between Rater’s B (second interview) and the site-independent rater and 29.4% were one point apart. Between the two site-based raters separately assessing and scoring the same subject on the same day, 69.4% of “dual” CGI-S scores at visit 1 were identical, and 31.6% were one point apart. The diagnosis of MDD or schizophrenia did not affect this proportion of inter-rater agreement.

At visit 2, 77.1% of “dual” CGI-S scores between site-based raters and the site-independent raters were identical, and 22.8% were one point apart.

### The SIGGI in MDD patients

There were 29 MDD subjects at visit 1 but only 6 at visit 2. This small MDD sample at visit 2 resulted from the random sampling design and some patient’s unwillingness to return without a new treatment intervention or other inducement. The first site-based rater administered the MADRS and the SIGGI, the second site-based rater administered the SIGGI only. Both SIGGI interviews were recorded with the audio-digital pen recorder and submitted to the blinded, site-independent rater for scoring. There were no significant CGI-S scoring differences between any raters at either visits 1 or 2.

The mean MADRS scores dropped by almost 9 points between the two visits. This marked change was primarily due to one of the six returning MDD patients whose MADRS score changed from 30 to 12 between visits. However, given the small sample size, there was still no statistically significant MADRS scoring difference between visits 1 and 2 in these 6 subjects (t = 0.99; df = 10; p = 0.35).

Within the MDD group, there was a significant correlation between the blinded site-independent CGI-S ratings with site-based rater 1 (r = 0.645; p =0.001) and rater 2 (r = 0.665; p = 0.001) at visit 1 and between the site-based CGI rater and site-independent rater at visit 2 (r = 0.840; p = 0.01).

Using a Pearson’s correlation, we compared the SIGGI derived CGI-S score with the MADRS as a validity measure in the MDD population at both visits. The CGI-S and MADRS scores were significantly correlated for all assessments (p = 0.001). At visit 1, the Pearson’s correlations was r = 0.554 with site-based rater 1, r = 0.745 with rater 2, and r = 0.576 with the site-independent rater. At visit 2, the correlations were r = 0.784 for the site-based CGI rater and r = 0.949 with the site-independent rater.

### The SIGGI and schizophrenia

42 patients with schizophrenia were seen at visit 1 and 29 returned for visit 2. Patients ranged in global illness ratings from borderline severity (CGI-S = 2) to moderately severe (CGI-S = 5). There were no significant mean CGI-S scoring differences between either of the two site-based raters or the site-independent rater on any CGI-S measure (Table 1).

Within the population of patients with schizophrenia, there was a significant correlation between the blinded site-independent CGI-S ratings with site-based rater 1 (r = 0.759; p =0.001) and rater 2 (r = 0.683; p = 0.001) at visit 1 and between the site-based CGI rater and site-independent rater at visit 2 (r = 0.818; p = 0.01).

Using the Pearson’s correlation for analysis, The SIGGI derived CGI-S score was compared with the PANSS as a validity measure in the patients with schizophrenia. The CGI-S and PANSS scores were significantly correlated for all assessments. At visit 1, the Pearson’s correlations was r = 0.566 with site-based rater 1, r = 0.577 with rater 2, and r = 0.793 with the site-independent rater. (p = 0.001 for all assessments). At visit 2, the correlations were r = 0.848 for the site-based CGI rater and r = 0.625 with the site-independent rater (p = 0.001 for both assessments).

## Discussion

We have developed a structured interview guide for global impressions (SIGGI) to assist in a more comprehensive documentation of the global impression assessment. The SIGGI provides specific queries to identify the relevant, acute symptoms, to assess the clinical relevance of these symptoms on behavior and function, and to consider the possible influence of confounding factors as well. The objective of this instrument is to improve the precision of CGI-S scoring within the context of good clinical judgment.

In this study, The SIGGI was both reliable and valid in the global assessment of patients with MDD and schizophrenia. High inter-rater reliability was demonstrated between site-based and blinded, site-independent raters as well as between the two site-based raters evaluating the patient on the same day. Comparison of SIGGI derived CGI-S scores with the validated symptom severity rating instruments of the MADRS and PANSS also revealed highly significant correlations between these measures.

The clinical global impression score is intended to be an expert clinical judgment that incorporates both the cumulative experience of the rater-clinician with the designated patient population and the documented information obtained about the patient’s current symptoms, behavior, and function. Rather than override that clinical judgment, the SIGGI fosters the generation of more precise data to support the derived scores.

The use of audio-digital pen recorders to allow the blinded, site-independent rating has been described elsewhere and has been shown to yield high correlations between site-based and site-independent raters [17]. Pen recordings have been used to verify diagnoses, confirm symptom severity, and assess interviewing competency [18]. Audio-recordings of site-based assessments for site-independent review can be a useful surveillance strategy to compare and confirm the reliability of global assessment scores in CNS trials. In this study, the recordings allowed for blinded reliability testing of the SIGGI without any informational variance because it used exactly the same information obtained by the site-based rater. The SIGGI queries provided sufficient documentation for blinded raters listening to a brief audio recording to produce CGI-S score that were highly concordant with the site-based raters. One limitation of this study is that we did not include a comparison group of CGI raters who did not administer the SIGGI. Therefore, we cannot compare the CGI-S scoring variance that would result with less recorded documentation.

## Conclusion

We have found that the SIGGI is a reliable and valid instrument that standardizes the documentation of the global severity of illness assessment. The CGI raters were usually able to administer the interviews within ten minutes. We believe that the SIGGI may have broad applicability in CNS clinical trials as a method to improve the documentation and precision of global ratings.

## Abbreviations

CGI-S: Clinical global impression of severity; CNS: Central nervous system; DSM-IV: Diagnostic and statistical manual- IV version; MADRS: Montgomery-asberg depression rating scale; MDD: Major depressive disorder; PANSS: Positive and negative syndrome scale; SIGGI: Structured interview guide for global impressions.

## Competing interests

Steven D. Targum

Acadia, Acumen, Acumen, Affectis, Alkermes inc., Amgen, AstraZeneca, BioMarin, BrainCells Inc., CeNeRx, Cephalon, Clintara LLC, CROnos, CTNI MGH, EnVivo Pharmaceuticals, Euthymics, Eli Lilly and Company, EnVivo Pharmaceuticals, Forest Research, Functional Neuromodulation inc, GlaxoSmithKline, Johnson & Johnson PRD, INC Research, Methylation Sciences Inc., NeoSync, Neurophage, Novartis Pharmaceuticals, Nupathe, Parexel International, PRA International, Prana Biotechnology Ltd., ReViva, Roche Labs, Sophiris, Sunovion, Targacept, Theravance, Transcept, Wyeth labs.

Celine Houser

Employee of Clintara LLC

Joanne Northcutt

Employee of Florida Clinical Research Center

Jessica A Little

Employee of Clintara LLC

Andrew Cutler

Founder/Director of Florida Clinical Research Center, Abbott, Acadia Pharmaceutical, Alkermes Inc, Arbor Scientia, AstraZeneca, Boehringer Ingelheim, BrainCells inc., Bristol Myers Squibb, CeNeRx, Cephalon, Cypress, Forest Labs, GlaxoSmithKline, Jazz, Johnson and Johnson PRD, Labopharm, Eli Lilly, Lundbeck, MedicoNova, Merck, Neuronex, NextWave, Novartis, Orexigen, Otsuka, PamLab, Pfizer Inc., Roche, Sanofi, Shionogi, Sunovion, Supernus, Targacept, Takeda, Theravance, UBC (Bracket), UCB, Vanda.

David Walling

Founder/Director of Collaborative Neuroscience Network, Abbott, Alkermes, Amgen, AstraZeneca, BrainCells Inc., Cephalon, Clintara LLC, Eisai, Forest, Johnson & Johnson PRD, Eli Lilly, Otsuka, Pfizr Inc., Shire, Sunovion, Takeda.

## Authors’ contribution

All authors listed made a substantial contribution to the design of this study, implementation of the research, and analysis of the data. In addition, SDT and JAL conducted the blinded review of audio-recorded interview, CH coordinated the process between trial sites, and JN, ALC, and DPW executed the studies and their respective clinical trial centers. All authors read and approved the final manuscript.

## Authors’ information

SDT is the Scientific Director and Founder of Clintara LLC and a consultant on the faculty at the Massachusetts General Hospital (Boston MA); CH and JAL are employees of Clintara LLC (Boston MA); JN and ALC are employees of Florida Clinical Research Center (Maitland FL), and DPW is founder/director of Collaborative Neuroscience Network 12772 Valley View Street, Suite 3 Garden Grove, CA 92845.

## Supplementary Material

Additional file 1 **Appendix 1.** Structured Interview Guide for Global Impressions (SIGGI). Clintara LLC, 2011. Click here for file

## References

[B1] Guy W ECDEU Assessment Manual for Psychopharmacology, Revised 1976 U.S. Department of Health, Education, and Welfare (DHEW) Publication ADM-76-338) 218 222

[B2] Kay SR Opler LA Fiszbein A The positive and negative syndrome scale (PANSS) for schizophrenia Schizophr Bull 1987 13 261 276 10.1093/schbul/13.2.261 3616518

[B3] Busner J Targum SD The clinical global impressions scale: applying a research tool in clinical practice Psychiatry 2007 4 7 28 37 20526405 PMC2880930

[B4] Targum SD Busner J Young AH Targeted scoring criteria reduces variance in global impressions Hum Psychopharmacol Clin Exp 2008 23 628 633 10.1002/hup.966 18666094

[B5] Goodman WK Price LH Rasmussen SA Mazure C Delgado P Heninger GR Charney DS The Yale-brown obsessive compulsive scale Arch Gen Psychiatry 1989 46 1012 1016 10.1001/archpsyc.1989.01810110054008 2510699

[B6] Spearing M Post RM Leverich GS Brandt D Nolen W Modification of the clinical global impression (CGI) scale for use in bipolar illness (BP): the CGI-BP Psychiatry Res 1997 73 159 171 10.1016/S0165-1781(97)00123-6 9481807

[B7] Schneider L Olin JT Doody RS Clark CM Morris JC Reisberg B Schmitt FA Grundman M Thomas RG Ferris SH Validity and reliability of the Alzheimer’s disease cooperative study-clinical global impression of change. The Alzheimer’s disease cooperative study Alzheimer Dis Assoc Disord 1997 11 Suppl 2 S22 S23 9236949 10.1097/00002093-199700112-00004

[B8] Haro JM Kamath SA Ochoa S The clinical global impression-schizophrenia scale: a simple instrument to measure the diversity of symptoms present in schizophrenia Acta Psychiatry Scandinavia 2003 107 Suppl 416 16 23 10.1034/j.1600-0447.107.s416.5.x 12755850

[B9] Levine SZ Rabinowitz J Engel R Etschel E Leucht S Extrapolation between measures of symptom severity and change: An examination of the PANSS and CGI Schizophr Res 2008 98 318 322 10.1016/j.schres.2007.09.006 17949948

[B10] Targum SD Hassman H Pinho M Fava M Development of a global impression scale for fatigue J Psychiatr Res 2012 46 370 374 10.1016/j.jpsychires.2011.12.001 22236834

[B11] Leon AC Marzak PM More reliable outcome measures can reduce sample size requirements Arch Gen Psychiatry 1995 52 867 871 10.1001/archpsyc.1995.03950220077014 7575107

[B12] Regier DA Kaelber CT Rae DS Farmer ME Knauper B Kessler RC Norquist GS Limitations of diagnostic and assessment instruments for mental disorders Arch Gen Psychiatry 1998 55 109 115 10.1001/archpsyc.55.2.109 9477922

[B13] Muller MJ Szegedi A Effects of interrater reliability of psychopathologic assessment on power and sample size calculations in clinical trials J Clin Psychopharmacol 2002 22 318 325 10.1097/00004714-200206000-00013 12006903

[B14] Sheehan DV Lecrubier Y Sheehan KH Amorim P Janavs J Weiller E Hergueta T Baker R Dunbar GC The Mini-International Neuropsychiatric Interview (M.I.N.I.): the development and validation of a structured diagnostic psychiatric interview for DSM-IV and ICD-10 J Clin Psychiatry 1998 59 suppl. 20 22 33 9881538

[B15] Kalali A Williams JBW Kobak KA Lipschitz J Engelhardt N Evans K Olin J Rotheman Pearson Bech P The new GRID HAM-D – Pilot testing and international field trials Int J Neuropsychopharmacol 2002 5 1 S147

[B16] Williams JBW Kobak KA Development and reliability of a structured interview guide for the Montgomery-Asberg Depression Rating Scale (SIGMA) Br J Psychiatry 2008 192 52 58 10.1192/bjp.bp.106.032532 18174510

[B17] Asgharnejad M Targum S Burch D Gibertini M Fava M Surveillance strategies to improve study outcomes in a depression study 2012 52nd Annual NCDEU meeting, Phoenix, Arizona

[B18] Targum SD Little JA Lopez E DeMartinis N Rapaport M Ereshefsky L Application of external review for subject selection in a schizophrenia trial J Clin Psychopharmacol 2012 32 2 825 826 10.1097/JCP.0b013e318248da90 22388170

[B19] Montgomery SA Asberg M A New depression scale designed to be sensitive to change Br J Psychiatry 1979 134 382 389 10.1192/bjp.134.4.382 444788

[B20] Rabinowitz J Levine S Martinez G Concordance between measures of functioning, symptoms, and change examining the GAF, CGI-S, CGI-C, and PANSS J Clin Psychopharmacol 2010 30 478 480 10.1097/JCP.0b013e3181e7145f 20631575

