# Supplementary material for: A structured interview guide for global impressions: increasing reliability and scoring accuracy for CNS trials
Source: Ann Gen Psychiatry. 2013 Jan 31;12:2. doi: 10.1186/1744-859X-12-2 (PMC3564924; doi:10.1186/1744-859X-12-2)
Supplement: Additional file 1 — Appendix 1. Structured Interview Guide for Global Impressions (SIGGI). Clintara LLC, 2011. [file 1744-859X-12-2-S1.pdf]

Structured Interview Guide for Global Impressions (SIGGI)

Clintara LLC, 2011

Instructions for Raters

This interview guide provides specific questions to fully explore the *global impact* of identified "targeted" symptoms of interest on behavior and function *within the past week*.

The objective of this instrument is to obtain the necessary documentation to support a derived global impressions score. Generally, the SIGGI interview *follows* the administration of a symptomatic questionnaire that identifies the key ("targeted") symptoms that may have *current* clinical relevance for the subject.

The interview proceeds as follows:

1. EXPLANATION: Begin the SIGGI by explaining the purpose of the interview and obtaining the subjects consent to participate.
2. SYMPTOM IDENTIFICATION: Inquire about (and/or confirm) the presence of relevant symptoms that have been present within the past week (PAST 7 DAYS).
3. IMPACT ASSESSMENT: Inquire about the amount of current (acute) distress or interference that the specific, "targeted" symptoms have caused for the subject.
4. IDENTIFICATION AND IMPACT OF CONFOUNDING FACTORS: Inquire about the presence and possible impact of confounding factors that may or may not have influenced behavior and/or function.
5. SCORE *AND* DOCUMENT: Using the scoring anchors provided, determine the most appropriate global score and provide written documentation to support the derived score.

1. EXPLANATION AND CONSENT

I'd like to ask you some questions about some of the symptoms that have experienced within the past week. Some of these questions may have already been asked and others will be new questions.

I'm interested in how much the symptoms that you've experienced in the past week have affected you at home, at work, at school, in your relationships with others, or while you pursue your usual interests or activities (such as hobbies).

Is that okay with you?

2. SYMPTOM IDENTIFICATION

Let's begin by listing the most troubling symptoms that you have experienced within the past week (7 days). Can you describe them?

How long have you had these symptoms (describe each specific symptom)?

SYMPTOM LISTING (Identify SYMPTOM and DURATION)

1. ______________________________ **Duration:** ____________________
2. ______________________________ **Duration:** ____________________
3. ______________________________ **Duration:** ____________________
4. ______________________________ **Duration:** ____________________
5. ______________________________ **Duration:** ____________________

NOTE TO RATER: Summarize and confirm each symptom and its duration before proceeding.

3. IMPACT ASSESSMENT

A. Have any of these symptoms interfered with your ability to function? ☐ **YES** ☐ **NO**

INQUIRE ABOUT RELEVANT ACTIVITIES: work, study, pursuit of hobbies or interests, participation in social events, or attending school.

IF SUBJECT RESPONDS YES, ASK***: In what way have the symptoms interfered?***
DOCUMENT RESPONSES IN THE SUBJECT'S OWN WORDS (with examples):

B. Have any of these symptoms interfered with your relationships? ☐ **YES** ☐ **NO**

INQUIRE ABOUT: friendships, social or work relationships, and interactions with family members.

IF SUBJECT RESPONDS YES, ASK***: In what way have the symptoms interfered?***
DOCUMENT RESPONSES IN THE SUBJECT'S OWN WORDS (with examples):

IF THE SUBJECT RESPONDS ***YES*** TO *EITHER* OF THE ABOVE IMPACT QUERIES (A or B), ASK THE FOLLOWING QUESTIONS:

Regarding these symptoms, how troubling or distressing have these symptoms been for you?

Can you give me an example?

DOCUMENT RESPONSES IN THE SUBJECT'S OWN WORDS (with examples):

QUANTIFICATION (RATER: refer to queries A or B above)
How many DAYS in the past week have these symptoms disturbed you or interfered with your behavior or function?
________________ days

How much of the time during the day (or night) have these symptoms disturbed you or interfered with your behavior or function? **________________ days**

Structured Interview Guide for Global Impressions

AWARENESS AND CONTROL

Do you think that others are aware of these symptoms or of the impact the symptoms have had on you in the past week? ☐ **YES** ☐ **NO**

Do you think you can mask or control the symptoms? ☐ **YES** ☐ **NO**

IF YES: ***How much?*** _______________ (Percent of time)

Have any of these symptoms caused you to completely stop doing any activity? ☐ **YES** ☐ **NO**

DOCUMENT RESPONSES TO THE ABOVE 3 QUESTIONS IN THE SUBJECT'S OWN WORDS (with examples):

RELEVANCE OF CURRENT SYMPTOMS: STATE versus TRAIT ASSESSMENT

This section assesses whether the identified symptoms are acute or long-standing symptoms and whether they have CURRENT clinical relevance.

AS A REFERENCE POINT FOR ASSESSING THE IMPACT OF THE CURRENT SYMPTOMS, IDENTIFY A TIME *PRIOR* TO THIS EPISODE WHEN THE SUBJECT FELT LIKE HIS/HER "NORMAL" SELF (felt okay).

Did you have these symptoms even when you felt your "normal" self (felt okay)? ☐ **YES** ☐ **NO**

Were any of your current symptoms present BEFORE the current episode? ☐ **YES** ☐ **NO**

Regarding these pre-existing symptoms, how long have they bothered you?

DOCUMENT RESPONSES IN THE SUBJECT'S OWN WORDS (with examples):

Are these symptoms more troublesome to you now, in the past week, than they were before your current episode? ☐ **YES** ☐ **NO**

DOCUMENT RESPONSES IN THE SUBJECT'S OWN WORDS (with examples):

4. IDENTIFICATION AND IMPACT OF CONFOUNDING FACTORS

The rater will identify and assess the presence and *possible* influence of any confounding factors on the global assessment score.

Since the beginning of this current episode (**STATE DURATION*****), or shortly before it began, did you experience any of the following:***

Relocating (moving) from another city, home, or living facility?	☐ **YES**	☐ **NO**
OR, did someone close to you move out of your home and away from you?	☐ **YES**	☐ **NO**
Getting married, giving birth, getting divorced (or separated from a loved one)?	☐ **YES**	☐ **NO**
Having a recent hospitalization?	☐ **YES**	☐ **NO**
Being in jail, being arrested, or involved in a lawsuit?	☐ **YES**	☐ **NO**
Having a serious medical illness in yourself or in someone close to you?	☐ **YES**	☐ **NO**
Having a change in any medication?	☐ **YES**	☐ **NO**
Having a change in your drinking or drug use?	☐ **YES**	☐ **NO**
Changing the doctor who has been treating your illness?	☐ **YES**	☐ **NO**
Experiencing a death of a close friend or relative?	☐ **YES**	☐ **NO**
Experiencing a traumatic experience, such as a robbery or accident?	☐ **YES**	☐ **NO**
Experiencing a very exciting experience, such as winning the lottery?	☐ **YES**	☐ **NO**

RATER: FOR ANY **YES** RESPONSE, DETERMINE IF THIS EXPERIENCE MAY HAVE INFLUENCED THE CURRENT SYMPTOMS AND/OR IMPACTED BEHAVIOR OR FUNCTION.

DOCUMENT RESPONSES IN THE SUBJECT'S OWN WORDS (with examples):

5. SCORE AND DOCUMENT
**CGI SEVERITY ASSESSMENT: _______________ (1-7)**<br>(use scoring guide/anchors provided for specific study/evaluation)<br><br>**DOCUMENT JUSTIFICATION FOR DERIVED SCORE FROM ALL AVAILABLE CLINICAL DATA:**
